# From data to action: a scoping review of wearable technologies and biomechanical assessments informing injury prevention strategies in sport

**DOI:** 10.1186/s13102-023-00783-4

**Published:** 2023-12-14

**Authors:** André Rebelo, Diogo V. Martinho, João Valente-dos-Santos, Manuel J. Coelho-e-Silva, Diogo S. Teixeira

**Affiliations:** 1 CIDEFES, Centro de Investigação em Desporto, Educação Física e Exercício e Saúde, Universidade Lusófona, 1749-024 Lisboa, Portugal; 2 COD, Center of Sports Optimization, Sporting Clube de Portugal, 1600-464 Lisbon, Portugal; 3 Research Unit for Sport and Physical Activity, Faculty of Sport Sciences and Physical Education, University of Coimbra, Coimbra, Portugal; 4 Laboratory of Robotics and Engineering Systems (LARSYS), Interactive Technologies Institute (ITI), Funchal, Portugal

**Keywords:** Athletic performance optimization, Biomechanical assessment, Recovery monitoring, Training load management, Wearable technology

## Abstract

**Background:**

The purpose of this scoping review was to evaluate the current use of technologies in sports settings for training adaptation and injury prevention. The review aimed to map the existing literature, identify key concepts and themes, and highlight gaps in research, thus offering guidance for future studies.

**Methods:**

This study followed the guidelines of the PRISMA extension for scoping reviews and a search in four major databases was conducted.

**Results:**

A total of 21 studies were included. The findings highlighted the widespread use of various technologies, including wearable devices and force plates, to monitor athletes’ performance and inform evidence-based decision-making in training and injury prevention. Variables such as Player Load, changes of direction, and acute chronic workload ratio were identified as key metrics in injury prediction.

**Conclusions:**

This review uncovers a dynamic field of research in athlete injury prevention, emphasizing the extensive use of varied technologies. A key finding is the pivotal role of Player Load data, which offers nuanced insights for customizing training loads according to sport-specific demands, player positions, and the physical requirements of various activities. Additionally, the review sheds light on the utility of tools like force plates in assessing fatigue, aiding recovery, and steering injury rehabilitation, particularly in sports prone to knee and ankle injuries. These insights not only enhance our understanding of injury prevention but also provide a strategic direction for future research, aiming to boost athlete safety, performance, and career longevity.

**Supplementary Information:**

The online version contains supplementary material available at 10.1186/s13102-023-00783-4.

## Background

The influence of load measurement, training adaptation, and injury prevention strategies in the context of sports and athletics has been a subject of extensive study in recent years, particularly in the impact that monitoring and analysing athletes’ training loads have on optimizing performance, reducing injury risks, and aiding in recovery processes [1, 2]. This has been fuelled by advances in sports monitoring technologies, including wearable devices such as accelerometers and global positioning system (GPS) tracking data, as well as non-wearable equipment like force plates. Simultaneously, the evolution in sports medicine and exercise science has seen the emergence of novel strategies to optimize training adaptation and prevent injuries [3].

The importance of monitoring athletes cannot be overstated in contemporary sports science. The proper quantification and management of training loads can facilitate optimal performance, reduce the risk of injury, and aid in recovery processes [4, 5]. Precise monitoring provides invaluable insights into an athlete’s physical condition, readiness for competition, and response to training interventions, thereby informing evidence-based decision-making in training programming [6].

With technological advancements, various monitoring tools have been developed and widely utilized in sports settings. Technologies, such as accelerometers, GPS trackers, and force plates, provide direct or indirect measures of athletes’ external and internal training loads. Accelerometers, for instance, measure acceleration forces, which can be used to estimate an athlete’s movements, energy expenditure, and even specific biomechanical patterns [7]. GPS trackers allow the tracking of athletes’ movement patterns, velocities, and distances covered, providing crucial information about their performance and physiological load during training or competition [8]. Force plates measure the ground reaction forces exerted by an athlete, offering insight into their biomechanics, strength, and power [9]. The data collected from these technologies can encompass various dimensions of an athlete’s performance. This includes their physical parameters such as speed, distance, and power, physiological parameters such as heart rate and metabolic rate, and biomechanical parameters such as ground reaction forces and movement patterns [10].

In the literature, various applications of these technologies have been reported. They have been used to evaluate the effectiveness of training programs, guide individualized training prescription, assess risk of injury, monitor recovery, and even provide real-time feedback to athletes and coaches [3]. Additionally, they are invaluable in rehabilitation settings, where they assist in assessing progress and readiness for return to play post-injury [11]. However, despite the wealth of information that these technologies provide, their integration into a holistic understanding of an athlete’s performance and health is a complex task, necessitating further comprehensive exploration.

The present review seeks to address this knowledge gap by conducting a scoping review of the literature. A scoping review was chosen over a systematic review due to its suitability for exploring broad, complex, and heterogeneous topics like the use of wearable technology in sports. Unlike systematic reviews, which focus on answering specific questions through rigorous data synthesis and analysis, scoping reviews are ideal for mapping a wide array of literature, identifying key concepts, and uncovering research gaps [12]. This approach is particularly valuable in emerging fields where literature is diverse and not yet ripe for comprehensive synthesis.

The research questions guiding this review include: What types of load measurement devices are currently being utilized in various sports for injury prevention? What are the key variables and outcomes that are commonly measured using these devices? How is the data from these devices integrated into training programs and injury prevention strategies across different sports?

## Methods

### Protocol and registration

The protocol for this scoping review was meticulously crafted to ensure thoroughness and consistency in the research methodology. The development process entailed comprehensive consultation of current literature, and relevant best practice guidelines, including the PRISMA extension for scoping reviews (Table S1 - Supplemental File), in order to formulate an effective strategy for carrying out the scoping review [13].

The protocol has been registered with the Open Science Framework (OSF) to ensure transparency and accessibility [14]. This step was taken as an assurance of the quality and reliability of the review process. The protocol was registered on the 14th of June 2023, and can be accessed freely online via the following link: https://osf.io/kyjv8.

### Eligibility criteria

The eligibility criteria for this scoping review were developed to guide the selection of relevant studies and ensure alignment with the research objectives. The focus of the review was conceptualized using the Population, Concept, Context (PCC) framework, recommended by the Joanna Briggs Institute for scoping reviews [15]. The Population refers to athletes involved in various sports. The Concept includes injury prevention, with an emphasis on the role of technologies in load measurement. The Context encompasses the conditions under which these measurements and strategies are applied, which can include various sports settings, training programs, rehabilitation scenarios, and risk management situations.

Population (P): This review encompasses studies involving athletes of all ages, including both male and female participants, across various levels of experience from amateur to professional. The rationale for including athletes from different levels is to capture a comprehensive understanding of how load measurement devices inform training and injury prevention strategies across different populations.

Concept (C): The review focuses on the utilization of load measurement devices in sport. This includes devices such as force plates, GPS systems, accelerometers, and wearable technology. The objective is to explore the existing literature on the use of these devices and how they contribute to injury prevention strategies in the field of sports.

Context (C): The review includes studies conducted in various sporting disciplines, including but not limited to team sports, individual sports, and endurance sports. The intention is to encompass a diverse range of sports contexts to allow for a comprehensive analysis of load measurement device usage across different athletic activities.

This review, while broadly encompassing a variety of study designs to capture diverse perspectives and methodologies, established specific exclusions to refine the focus. Therefore, review articles, meta-analyses, opinion pieces, and case reports were not included. This exclusion criteria were carefully chosen to ensure the inclusion of original research studies that provide direct evidence on the use of load measurement devices in sports for injury prevention. The review only included English language publications to ensure feasibility in the analysis and synthesis of the available literature. There were no restrictions on the publication date to capture a wide range of relevant studies.

### Information sources

In the pursuit of comprehensiveness and inclusivity, the present review’s search strategy involved an extensive examination of published literature. Four major databases were utilized to ensure broad coverage: PubMed/Medline, Scopus (Elsevier), Web of Science (Clarivate), and SPORTDiscus (EBSCOhost). These databases were selected to offer a balance between biomedical, psychological, and sports science perspectives. The literature search in these databases was last executed on June 15, 2023.

To supplement the database search, the present review also adopted additional search methods. These included scanning the reference lists of pertinent reviews and articles for additional sources that might not have been identified in the initial database search. This process, commonly known as “snowballing”, can help capture relevant studies that might otherwise be overlooked. Key journals in the field were also hand-searched to uncover articles of relevance that may not have been indexed in the databases used. This comprehensive and systematic search strategy was designed to ensure that all relevant studies were identified, regardless of their publication status or source.

### Search

The search strategy developed for the present review was meticulously designed and carried out to promote easy replication by other researchers. Additionally, the search strategy followed the Peer Review of Electronic Search Strategies (PRESS) checklist to ensure the robustness and comprehensiveness of the search [16].

The search was conducted using a combination of Medical Subject Headings (MeSH) terms and keywords in titles and abstracts. The strategy was designed to capture articles pertaining to load measurement, force plate, accelerometer, GPS tracking, wearable technology, athletes, sports, training load, exercise, training adaptation, overtraining, injury prevention, risk management, and rehabilitation. The entire search strategy for each database is available in the supplemental material file.

The review employed specific search limitations to refine the scope of retrieved results. Filters such as “Journal Article” for publication type and “English” for language were applied to facilitate manageability and relevance of the search results. These limitations were deemed necessary for the practical execution of the review and to ensure the quality and relevance of the articles included.

### Selection of sources of evidence

The process of selecting sources of evidence for this review involved a comprehensive, multi-step screening procedure to ensure the relevance and quality of the studies included. The process began with title screening (A.R. and D.V.M.), which involved a systematic review of the titles of retrieved articles to exclude any that were patently irrelevant to the review’s focus. Subsequently, an abstract and keyword screening was conducted on the remaining articles (A.R., D.V.M., and D.S.T.), assessing their relevance based on the inclusion and exclusion criteria.

To standardize the selection process, a review form was developed, containing pertinent questions about the study design, methodology, and findings. The form was developed and tested iteratively within the review team, with adjustments made based on feedback from initial testing. A software platform, CADIMA, was employed to facilitate the title screening and deduplication procedures, thus ensuring a systematic and consistent process [17].

Prior to the official screening, a calibration exercise was undertaken to validate the screening form and process. A subset of citations and full-text articles was independently screened by the review team members. Discrepancies were identified and discussed, leading to further refinement of the form and clarification of the screening criteria.

In the full screening process, each citation and full-text article was independently assessed by two reviewers. Any discrepancies between the reviewers were resolved through discussion and, if necessary, referral to a third reviewer for a final decision. A verification process was implemented to ensure that all relevant studies were included, and duplicates were identified and removed using CADIMA to maintain the integrity of the review. This rigorous selection process was employed to ensure the comprehensiveness and relevance of the studies included in the review.

### Data charting

The data charting process for the present review was underpinned by a carefully designed form, utilized to extract the most salient information from the selected sources of evidence. The items charted encompassed variables of interest, estimations of associations or effect sizes, qualitative data fragments, descriptions of methods, and metadata. The selection of these items was guided by their relevance to the research questions.

To ensure the consistency and reliability of the data charting, a calibration exercise was conducted. The review team members independently charted a subset of sources using the initial version of the charting form. The team then compared their charting results and resolved discrepancies through discussion. These discussions facilitated the identification of necessary changes or refinements to the charting form and procedure.

The full charting process was implemented by multiple reviewers, with each source being independently charted by at least two reviewers. Any inconsistencies between reviewers in their charting were resolved through discussion or, where necessary, the input of a third reviewer. This approach ensured the thoroughness and accuracy of the charting process. Changes made to the charting form during the iterative process were carefully documented, along with the rationales for those changes. This documentation ensures transparency and allows for replication and further refinement in future reviews.

For data that were unclear or missing from the source documents, attempts were made to contact the original investigators for clarification or additional information. The obtained data were then cross-verified with the published sources to confirm their accuracy and validity. This rigorous process was critical to ensuring the integrity of the data used in the review.

### Data items

For the current review, data items were carefully selected to address the study’s focus on the use of load measurement devices, training parameters, injury prevention strategies, and rehabilitation protocols for athletes.

Certain items involved interpretation, such as the training adaptation, overtraining, and rehabilitation strategies. For these items, charting required a detailed reading and understanding of the source document to accurately represent the study’s methodologies and outcomes. Details about the methods of included studies, such as study design, sample size, sample characteristics, and timing of data collection, were also charted. These pieces of information were crucial for contextualizing the findings and assessing their applicability to the research questions. Lastly, metadata including author names, institutions, year of publication, and journal or source of publication were charted for each source.

### Critical appraisal of individual sources of evidence

Given the nature of the present scoping review, a critical appraisal of individual sources of evidence was not a primary focus. The main aim was to map the existing literature and identify key concepts, sources of evidence, and research gaps related to load measurements, training adaptation, and injury prevention in athletes. Therefore, the review did not attempt to synthesize and weigh the evidence to draw conclusions or make recommendations, which is more typical of systematic reviews.

However, to ensure the general credibility of the sources included, a basic quality check was conducted. This consisted of evaluating the relevance of each study to the research questions and assessing whether the methodologies used in the studies were sufficiently rigorous and clearly described. This process was performed by the review team during the data charting phase.

It is important to clarify that the findings from this step did not influence the inclusion of sources in the review or the extraction of data. All sources that met the inclusion criteria and provided relevant data were included, regardless of their individual quality ratings. The intent was to capture a broad scope of the literature in the field, including potentially differing perspectives and methodologies.

### Synthesis of results

The synthesis of results in the present review was structured to systematically capture and present the breadth of evidence related to load measurements, training adaptation, and injury prevention in athletes. The primary aim was not to draw definitive conclusions, but to map the range of evidence and identify key concepts, common themes, and gaps in the research field.

The synthesis process involved grouping the extracted data into thematic clusters based on their relevance to the research objectives. Each theme was then explored and summarized narratively, with the purpose of providing an overview of the existing literature in that area and pointing out any significant findings, trends, consistencies, or divergences.

In addition to the narrative synthesis, the results were also presented visually. This involved creating tables that provide an at-a-glance understanding of the key findings and themes. For instance, a summary table was used to display key information about each included study, such as the variables measured and the main findings.

## Results

### Selection of sources of evidence

**Fig. 1:**
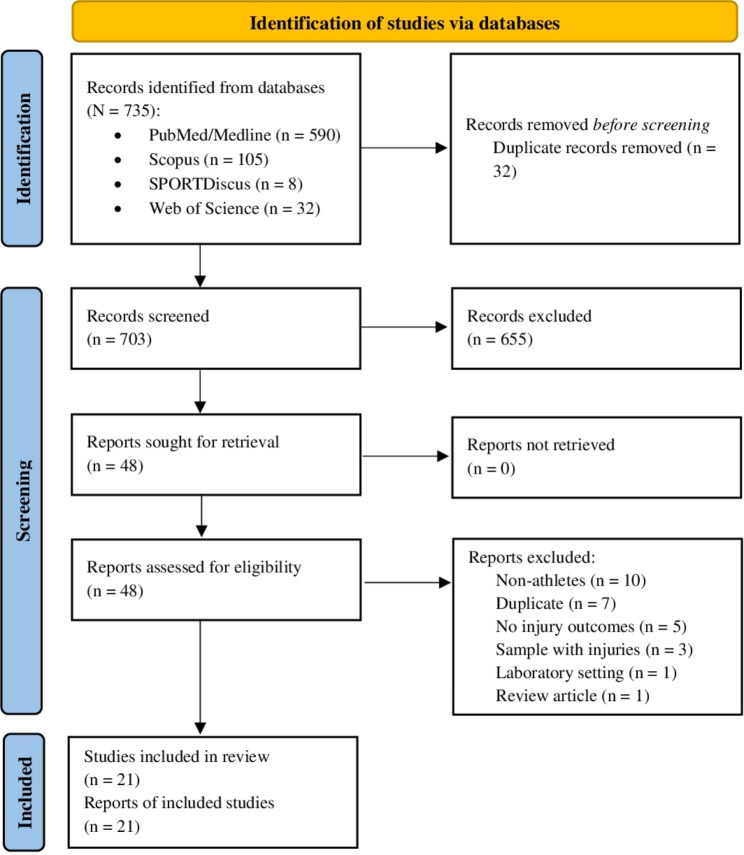
PRISMA flow diagram

The search strategy identified a total of 735 records across PubMed/Medline (n = 590), Scopus (n = 105), SPORTDiscus (n = 8), and Web of Science (n = 32). After removing 32 duplicates, 703 records were screened, leading to the exclusion of 655 records for lack of relevance. The remaining 48 full-text articles were further assessed, resulting in the exclusion of several reports due to various reasons (non-athletes (n = 10), duplicates (n = 7), lack of injury outcomes (n = 5), inclusion of athletes with pre-existing injuries (n = 3), conducted in a laboratory setting (n = 1), and review article (n = 1)), ultimately leaving 21 studies that met the inclusion criteria and were included in this scoping review. The selection process is illustrated in Fig. 1.

### Characteristics of sources of evidence

A total of 21 studies were included in the scoping review, with participant numbers ranging from 11 to 232 across these studies [18–38]. These studies represented a varied demographic of athletes, with ten studies (48%) focusing exclusively on male athletes [23, 25, 26, 29, 30, 32, 34–37], eight studies (38%) focusing on female athletes [18, 20–22, 24, 27, 28, 38], and three studies (14%) including both male and female participants [19, 31, 33]. The age of participants spanned a wide range from 16 to 42 years. The majority of the studies (n = 10; 48%) included collegiate athletes [18, 21, 22, 24–26, 28, 33, 35, 38], followed by professional athletes (n = 6; 29%) [23, 29, 30, 32, 34, 36]. The sports represented were diverse, with six studies (29%) focusing on soccer players [19, 22, 27, 30, 33, 38], five (24%) on volleyball players [18, 21, 24, 28, 33], four (19%) on American football players [23, 25, 26, 35], and four (19%) on Australian football players [29, 32, 34, 36]. Interestingly, a third of the studies (33%) included indoor sports [18, 20, 21, 24, 28, 33, 38]. This information can be seen in Table 1.

**Table 1:** Descriptive data of the included studies

Study	N	Sex (M/F)	Age	Height (cm)	Weight (kg)	Level	Sport
Augustsson & Andersson, 2023	15	M (n = 12) and F (n = 3)	19 ± 3	182 ± 8	74 ± 10	Elite	Soccer and track and field
Schmidt et al., 2023	25	F	16.3 ± 1.2	172 ± 6	70 ± 10	Elite	Handball
Taylor et al., 2022	16	F	19.6 ± 1.1	178 ± 7.0	75.9 ± 9.3	Collegiate	Volleyball
Kupperman et al., 2021	32	F	20 ± 1	168.75 ± 4.28	Not reported	Collegiate	Soccer
Kupperman et al., 2021	11	F	19.36 ± 1.27	169.91 ± 3.88	64.04 ± 7.08	Collegiate	Volleyball
Li et al., 2020	232	M	Not reported	Not reported	Not reported	Professional	American football
Sinsurin et al., 2020	19	F	19.7 ± 1.4	Not reported	Not reported	Collegiate	Volleyball
Sanders et al., 2020	40	M	20.5 ± 1.4	188.6 ± 3.3	107.1 ± 5.0	Collegiate	American football
Greig et al., 2019	22	F					Soccer, rugby, and field hockey
Murray et al., 2019	63	M	20.6 ± 1.5	186 ± 7.7	102.4 ± 20.1	Collegiate	American football
Rossi et al., 2018	26	M	26 ± 4	179 ± 5	78 ± 8	Professional	Soccer
Stiles et al., 2018	35	M (n = 16) and F (n = 19)	41.9 ± 11.4	172 ± 8.0	68.5 ± 9.7	Not reported	Long distance running
Rostami et al., 2018	32	F	20.66 ± 1.1	170.5 ± 2.4	57.4 ± 5.1	Collegiate	Volleyball
Garrett et al., 2018	23	M	P: 22.5 ± 4.2 SP: 22.3 ± 2.9	P: 190.1 ± 6.5 SP: 184.4 ± 5.8	P: 87.4 ± 6.8 SP: 80.9 ± 6.2	Professional and semiprofessional	Australian football
Carey et al., 2017	75	M	Not reported	Not reported	Not reported	Professional	Australian football
Liu et al., 2016	61	M (n = 29) and F (n = 32)	19.9 ± 1.2	176.6 ± 9.5	74.3 ± 10.8	Collegiate	Basketball, soccer, baseball, softball, tennis, and volleyball
Wilkerson et al., 2016	45	M	20.1 ± 1.3	187.4 ± 5.7	104.1 ± 15.6	Collegiate	American football
Ritchie et al., 2016	44	M	24.1 ± 3.8	187.7 ± 7.2	87.3 ± 8.2	Professional	Australian football
Colby et al., 2014	46	M	25.1 ± 3.4	188.0 ± 6.8	87.0 ± 8.2	Professional	Australian football
McLellan et al., 2011	17	M	19.0 ± 1.3	188 ± 2.3	89.6 ± 15.8	Elite	Rugby league
Chappell & Limpisvasti, 2008	30	F	19 ± 1.2	174 ± 8.5	69.8 ± 10.9	Collegiate	Basketball and soccer

With regards to the methodological considerations and study settings (Table 2), nine studies (43%) were conducted solely during the competitive phase (in-season) [18, 20, 25, 26, 29, 30, 32, 35, 37], while six studies (29%) spanned both the preparatory (pre-season) and competitive phases [21, 23, 31, 34, 36, 38]. A single study (5%) was conducted during the preparatory phase only [22] and another one (5%) during the transition phase only [19]. Four studies (19%) did not specify the phase of the season in which they were conducted [24, 27, 28, 33]. In terms of equipment used to measure and monitor athletes, a significant number of studies incorporated triaxial accelerometer units (n = 14; 67%) [18, 21–23, 25–27, 29–32, 34–36], while GPS technology was included in 11 studies (52%) [22, 23, 25–27, 29, 30, 32, 34–36]. Seven studies (33%) utilized force plate assessments in their methodologies [19, 20, 24, 28, 33, 37, 38].

**Table 2:** Methodological considerations and study settings of the included studies

Study	Duration of the study	Training phase	Type of device(s)	Validity and reliability of device reported	Methodological considerations
Augustsson & Andersson, 2023	One day	Transition (off-season)	Linear encoder, load cell and force plate	Not reported	Sample rate was 200 Hz and no filter was used.
Schmidt et al., 2023	12 weeks	Competitive (in-season)	Force plate and a three-dimensional motion capture system consisting of 12 infrared cameras	Not reported	Sample rate was 1000 Hz and 120 Hz for the force plate and the three-dimensional motion capture system, respectively. Data were filtered with a fourth-order digital Butterworth filter with a cutoff frequency of 20 Hz.
Taylor et al., 2022	One competitive season	Competitive (in-season)	Triaxial accelerometer unit	Authors reported the validation and reliability of the variables measured according to MacDonald et al., 2016.	Each player wore the unit in an elastic waistband just inferior and lateral to their umbilicus. Each device transmitted data through Bluetooth technology to a portable tablet.
Kupperman et al., 2021	Three months	Preparatory (pre-season)	GPS and triaxial accelerometer unit	Good accuracy and reliability were reported according to Boyd et al., 2011 and Johnston et al., 2012	Sampling rates of 10 and 100 Hz for GPS and accelerometer, respectively.
Kupperman et al., 2021	18 weeks	Preparatory (pre-season) and competitive (in-season)	Triaxial accelerometer unit	Excellent intradevice reliability with ICCs ranged from very large to nearly perfect according to Nicolella et al., 2018.	The units were sampled at a rate of 100 Hz.
Li et al., 2020	Two seasons. Each with 14 weeks pre-season + 17 weeks in-season	Preparatory (pre-season) and competitive (in-season)	GPS and triaxial accelerometer unit	Authors reported the validation of the variable measured according to Nicolella et al., 2018.	Sampling rates of 15 and 100 Hz for GPS and accelerometer, respectively.
Sinsurin et al., 2020	One day	Not reported	Force plate and a motion capture system consisting of 10 infrared cameras	Not reported	Sample rate was 1000 Hz for the force plate. Data were filtered using a fourth-order zero-lag Butterworth digital filter at cut-off frequencies of 6 and 40 Hz, for the motion capture system and force plate, respectively.
Sanders et al., 2020	One competitive season	Competitive (in-season)	Wearable microsensor that included a GPS, gyroscope, magnetometer and triaxial accelerometer with an inertial movement sensor	Validity and reliability were reported according to Cummins et al., 2013 and Luteberget et al., 2017	The wearable microsensor device worn included a 10 Hz GPS, 100 Hz gyroscope, 100 Hz magnetometer, and 100 Hz triaxial accelerometer with inertial movement analysis technology.
Greig et al., 2019	One day	Not reported	GPS and triaxial accelerometer unit	Not reported	Triaxial acceleration data was collected at 100 Hz.
Murray et al., 2019	One full competitive season	Competitive (in-season)	Inertial measurement units containing GPS and a triaxial accelerometer unit	Not reported	Triaxial acceleration data was collected at 100 Hz.
Rossi et al., 2018	23 weeks	Competitive (in-season)	GPS and triaxial accelerometer, gyroscope, and digital compass	Not reported	Sampling rates of 10 and 100 Hz for GPS and triaxial units, respectively.
Stiles et al., 2018	6 months	Preparatory (pre-season) and competitive (in-season)	Triaxial accelerometer	Not reported	Triaxial acceleration data was collected at 100 Hz.
Rostami et al., 2018	6 weeks	Not reported	Force plate	Not reported	The data was recorded at a sampling rate of 250 Hz and within 8 s. Then, using the Butterworth 4th grade low-pass filter, the force plate data were filtered.
Garrett et al., 2018	1 week	Competitive (in-season)	Optical encoder, GPS and triaxial accelerometer unit	Authors reported the reliability of GPS-embedded triaxial accelerometers according to Aughey., 2011 and Cormack et al., 2013	Triaxial acceleration data was collected at 100 Hz.
Carey et al., 2017	3 seasons	Competitive (in-season)	GPS and triaxial accelerometer unit	Validity was reported according to Luke et al., 2011 and Rampinini et al., 2015	Sampling rates of 10 and 100 Hz for GPS and accelerometer, respectively.
Liu et al., 2016	Not reported	Not reported	Force plate	Not reported	Force plate data were collected at a sampling rate of 100 Hz.
Wilkerson et al., 2016	15 weeks	Competitive (in-season)	Inertial measurement units containing GPS and a triaxial accelerometer unit	Validity was reported according to Boyd et al., 2011 and Gabbett et al., 2013	Acceleration data was collected at 100 Hz.
Ritchie et al., 2016	One season	Preparatory (pre-season) and competitive (in-season)	GPS and triaxial accelerometer unit	Validity was reported according to Rampinini et al., 2015	Sampling rates of 10 and 100 Hz for GPS and accelerometer, respectively.
Colby et al., 2014	One season	Preparatory (pre-season) and competitive (in-season)	GPS and triaxial accelerometer unit	Validity was reported according to Jennings et al., 2010 and Johnston et al., 2012	Sampling rates of 15 and 100 Hz for GPS and accelerometer, respectively.
McLellan et al., 2011	One game	Competitive (in-season)	Force plate	Not reported	Sample rate was 1000 Hz for the force plate. The vertical force–time data were filtered using a fourth-order Butterworth low-pass filter with a cutoff frequency of 17 Hz.
Chappell & Limpisvasti, 2008	6 weeks	Preparatory (pre-season) and competitive (in-season)	Force plate	Not reported	Sample rate was 2400 Hz for the force plate.

### Results of individual sources of evidence

From the collected evidence, it became clear that the majority of studies (n = 12; 57%) preferred to collect data during actual training sessions and/or matches [18, 21–23, 25, 26, 30–32, 34–36]. Player Load, which is a measure of an athlete’s workload defined as the instantaneous rate of change of acceleration divided by a scaling factor [39], was used as a primary outcome in nearly half of the studies (n = 10; 48%), collected by wearable technologies including GPS devices and accelerometers [21–23, 25–27, 29, 32, 34, 35]. This external training load data was found to influence decisions made by coaches and practitioners in regard to injury prevention (Table 3).

**Table 3:** Results and practical applications for training

Study	Test(s)	Variables measured	Impact on injury prevention decisions
Augustsson & Andersson, 2023	Nordic hamstring curl (eccentric only and eccentric-concentric). Peak force at the ankle, peak force about the knee joint and forward distance achieved.	Peak force at the ankle, peak force about the knee joint and forward distance achieved.	The eccentric-concentric variation involves movement at shorter hamstrings length, and it may be more well tolerated by an athlete during hamstring injury rehabilitation and a less strenuous alternative for a novice athlete.
Schmidt et al., 2023	Double-leg drop vertical landing (DLL), single-leg drop landing (SLL), and an unanticipated side-cutting task.	Knee flexion angle, internal rotation angle, knee abduction angle, and vertical ground reaction force	Both devices were useful in capturing variables that might have influence in detecting biomechanical risk factors associated with ACL injury risk. However, the use of a three-dimensional motion capture system might be unrealistic in most youth clubs settings.
Taylor et al., 2022	Variables were collected during the specific sport training sessions and games	Jump count, average jump height, maximum jump height, and jump load	Those athletes who were injured performed significantly fewer jumps per athletic exposure and had larger variation in external training loads before their injury.
Kupperman et al., 2021	Variables were collected during the specific sport training sessions and games	Total distance and Player Load	Coaches could use this information to assess each player by their position on the field. Furthermore, each different drill had distinct physical demands. Defenders had the highest overall demand during practices. Across all positions, simulated game-play data imposed the highest load on athletes, while tactical skills drills had higher Player Load intensity. Practitioners can use these results during the pre-season period to prescribe training loads individualized to each drill and playing position.
Kupperman et al., 2021	Variables were collected during the specific sport training sessions and games	Player Load, changes of direction, accelerations, decelerations, repeated high-intensity efforts, and jumps.	Player Load and changes of direction can reflect better the activity in all positions in volleyball. These accelerometers can, therefore, be a valuable addition to a volleyball team setting as they can measure different variables that can evaluate the positional differences among players.
Li et al., 2020	Variables were collected during the specific sport training sessions	Player Load	A greater proportion of injuries are associated with higher levels of acute chronic workload ratio over 1.6. During the in-season, injury was associated with overall lower training workloads in the week prior to injury. These findings suggest that the use of GPS and accelerometers in professional American football can be useful to evaluate the training loads of these athletes.
Sinsurin et al., 2020	Jump landings in multiple directions	Angular velocity, flexion excursion, and vertical ground reaction force	The protocol may be useful in detecting knee coordination levels in female volleyball athletes that are at higher risk of knee injury.
Sanders et al., 2020	Variables were collected during games	Player Load, accelerations, decelerations, and changes of direction	The results highlight the importance to monitor and potentially train for maximal workloads to fully prepare athletes for the rigors of competition. The results also support the specific training load prescriptions based on player positions. Practitioners can use this data during the rehabilitation process to meet the requirements of the game. Finally, athletes reported no issues of wearing the device during the games.
Greig et al., 2019	Anterior hop, inversion hop, eversion hop, diagonal hop, and diagonal hurdle hop	Player Load	This study advocates a placement closer to the anatomical rehabilitation site of interest. The triaxial accelerometery (embedded within GPS technology) showed promising results in assessing Player Load in different hop tests in athletes.
Murray et al., 2019	Variables were collected during the specific sport training sessions and games	Stride variability and Player Load	This study has shown that stride variability is associated with fatigue and 7-day training load. This study highlighted that is possible to identify individual athletes who have an elevated period of load compared to their normal training load even when there is data missing. This can be accomplished through the accelerometer data of stride variability. This can be useful for coaches working with team sport athletes during long periods, as they can establish baseline values to then assess their athletes’ injury risk.
Rossi et al., 2018	Variables were collected during the specific sport training sessions and games	Distance covered, accelerations, decelerations, and impacts.	Practitioners should take particular care of the first training sessions of players who come back to regular training after a previous injury, as in these conditions they are more likely to get injured again. In these first days and in the days long after the players return to regular physical activity, the club should control kinematic workloads
Stiles et al., 2018	Variables were collected during the specific sport training sessions	Average acceleration and minutes above 400 milligravitational units	The use of a wrist accelerometer removes the reliance on the creation of a subjective training log, reduces participant burden, avoids bias, and facilitates the accurate monitoring of runners training behaviour. There was a high monitor wear compliance which shows that this device is useful in reporting metrics associated with training loads in runners which might give valuable insights for coaches.
Rostami et al., 2018	Stick landing and step back landing	Ground reaction force, rate of loading, and dynamic postural stability index	This study showed that a force plate system might be useful to measure kinetic variables that have influence in reducing the risk of ACL injuries in female volleyball athletes. Coaches can use these methods during a return-to-play scenario or to assess which athletes are at higher risk of sustaining an injury.
Garrett et al., 2018	Countermovement jump and submaximal run	Jump height and Player Load	The results show selected outcome triaxial accelerometer variables of a submaximal run can be used to assess neuromuscular fatigue in Australian football athletes. The ability to be administered as part of the warm-up or immediately postgame, to a large group of athletes can allow valuable information on recovery status and injury risk.
Carey et al., 2017	Variables were collected during the specific sport training sessions and games	Player Load, distance, and moderate and high speed running	Hamstring injury models showed potential for better performance than general non-contact injury models. This study showed that Australian football athletes’ might benefit from using a GPS and a triaxial accelerometer daily.
Liu et al., 2016	Forward hop and lateral hop	Time to stabilisation	This study was able to distinguish among healthy and unstable ankles of collegiate athletes. This shows that a force plate might be a useful device for coaches that work with athletes that are at higher risk of sustaining ankle injuries.
Wilkerson et al., 2016	Variables were collected during the specific sport training sessions and games	Player Load	Both accumulated inertial load and low variability in average inertial load seem to be important indicators of elevated risk for injury among contact sport athletes. Data derived from these devices may ultimately prove to be exceptionally valuable for guidance of individualized performance enhancement, injury prevention, and injury rehabilitation program design in team sport contact athletes.
Ritchie et al., 2016	Variables were collected during the specific sport training sessions and games	Total distance, high-speed running, mean speed, and Player Load	This study showed that the concept of “train as you play” is highly impractical due to the high game demands and increased injury risk. Coaches can use GPS devices with Australian football to manage loads during the weeks to reduce the injury risk.
Colby et al., 2014	Variables were collected during the specific sport training sessions and games	Distance, sprint distance, velocity load, and relative velocity change	Derived running loads should be regularly monitored, as they may significantly relate to player injury risk. The specific loads identified in this study provide guidelines for the volumes that should be considered in Australian football for representing increases in injury risk. In a practical sense, load thresholds might then be determined for individual players, above which injury risk substantially increases. Implementing both GPS and accelerometer devices in a professional Australian football club setting may provide useful information regarding injury risks.
McLellan et al., 2011	Countermovement jump	Peak rate of force development, peak power, and peak force	The present study found that a return to modified strength training activities 48 h post-match may result in a compensatory increase in peak rate of force development and supports the early implementation of strength training methods to facilitate the short-term post-match recovery period. This study highlights the importance of having a force plate to measure force and power variables in team-sport setting to make informed decisions about injury and training specific programs.
Chappell & Limpisvasti, 2008	Drop jump and vertical stop jump	Peak vertical ground reaction force and time on force plate	The results from this study support the use of a force plate in female soccer and basketball players to reduce their injury risk. This device can be used during the pre-season and in-season phases.

In each sport, the focus of data collection and interpretation varied. In soccer, Player Load data was used to assess players based on their positions on the field [22, 27]. In volleyball, Player Load and changes of direction were key parameters that could reflect the activity of all players across positions [21]. American football studies highlighted the acute chronic workload ratio as a key metric in injury prediction [23, 25, 26, 35]. Studies with female athletes promoted the use of hops and jumps tests to monitor injury risk [20, 24, 27, 28, 33, 38]. Lastly, in Australian football, the use of GPS and accelerometers for daily monitoring was a common practice, and triaxial accelerometer variables from submaximal runs were used to assess injury risk [29, 32, 34, 36].

### Synthesis of results

The synthesis of results, not only from studies that focused on Player Load, but also from those that investigated other variables, illustrates the broad and critical impact of different devices in sports science. Depending on the specific sport and research question, various technologies such as GPS, accelerometers, and force plates, among others, were used.

In studies involving soccer and track and field athletes, the use of force plates, coupled with specific testing protocols like the Nordic hamstring curl, allowed for improved understanding of muscular tolerances during injury rehabilitation [19]. In female handball and volleyball athletes, force plates and motion capture systems helped in detecting biomechanical risk factors associated with ACL injury risk [20, 24]. However, some studies highlighted the practical limitations of using a three-dimensional motion capture system in youth club settings [20].

In professional soccer, integrating GPS, accelerometer, gyroscope, and digital compass data led to more effective workload management during injury recovery phases. Similarly, the use of a wrist accelerometer in long-distance runners provided an objective, accurate method for monitoring training behaviour, thus reducing reliance on subjective training logs [31].

In collegiate sports involving both male and female athletes, such as basketball, volleyball, softball, soccer, tennis, and baseball, force plate systems proved valuable in distinguishing between healthy and unstable ankles, aiding in identifying athletes at a higher risk of sustaining ankle injuries [33].

In elite rugby league players, force plate measurements provided insights on the benefits of modified strength training activities in facilitating short-term post-match recovery [37]. Similarly, in female basketball and soccer players, force plate measurements supported injury risk reduction and could be applied in both pre-season and in-season phases [38].

## Discussion

### Summary of evidence

In this scoping review, 21 primary studies on injury prevention in athletes, employing various types of load measurement devices, were examined. The studies, published between 2008 and 2023, offered a broad perspective on the current practices across an array of sports, including soccer, volleyball, and both American and Australian football.

Findings from these studies highlighted the rising emphasis on the use of technology in monitoring and managing athletes’ workload. Notably, triaxial accelerometer units featured in two-thirds (67%) of the studies, illustrating their crucial role in injury prevention. Furthermore, over half of the studies (52%) integrated GPS technology, while one-third (33%) utilized force plates, demonstrating a wide application of diverse technologies to gather invaluable data during training and matches. This data, in turn, informed decisions made by coaches and practitioners regarding injury risk, underscoring the growing importance of athlete monitoring. Monitoring equips practitioners with a wealth of objective data, enabling them to tailor training programs, optimize recovery, and prevent injuries. In a field where overuse and fatigue can lead to significant injuries, these technologies serve as an early warning system, guiding modifications to training regimes as necessary.

The fatigue continuum is an invaluable concept that can offer key insights into managing athlete health and performance. It describes a spectrum of states through which athletes can transition, ranging from being well-recovered to overtrained [40]. Understanding that fatigue and recovery are dynamic, continuous processes becomes pivotal in designing and adjusting training regimes accordingly to maintain optimal performance levels and mitigate potential injuries. By integrating load monitoring devices into training regimens, practitioners can track and quantify an athlete’s physiological responses and workloads [41]. These devices can provide critical data on workload parameters such as distance covered, speed, acceleration, and impacts, among others, allowing for an objective assessment of fatigue levels [42]. With this information, practitioners can identify early signs of excessive fatigue that could potentially lead to overtraining or injuries [40]. As such, the real-time and longitudinal data these devices provide can guide decisions on when to push harder or when to hold back in training, helping athletes to stay in the well-recovered end of the continuum. This approach allows the effectiveness of training to be optimized while ensuring the athlete is not pushed into a state of overreaching or overtraining, both of which heighten the risk of injury [40].

The review also highlighted an increasing focus on comprehensive, multifactorial assessment of athlete load in injury prevention research, with Player Load emerging as a commonly measured variable in 48% of the studies. Moreover, the integration of these monitoring strategies with coaching was evident across the studies. Real-time, situational data collected during training sessions and matches informed coaching decisions, suggesting an emphasis on integrating scientific data with practical coaching. This approach allows for more individualized training plans and more effective management of player loads, further emphasizing the importance of these devices in contemporary sports practices.

Effective athlete monitoring, as evidenced in this study, is not without its challenges. Several barriers were identified in implementing these technologies, each necessitating its own unique approach to overcome. A primary challenge is the need for proper training in data interpretation. It emerged that the successful implementation of monitoring strategies relies heavily on the proper use of statistical tools [43]. Practitioners often employ basic statistical analyses such as mean, standard deviation, and percentage change, to make sense of the gathered data and make informed decisions on training adjustments. These tools help identify patterns, highlight outliers, and track changes over time, allowing for proactive injury prevention strategies [43]. However, this review also highlighted that only nine studies (43%) reported the validity and/or reliability of their devices used. The reliability of the monitoring tools is often considered the most important factor because it affects the precision of the monitoring of athletes [44]. This skill gap can be addressed through ongoing professional development and education. Workshops, seminars, or even online courses can equip sports professionals with the necessary skills to interpret and apply data. Additionally, technology developers could also provide more user-friendly software with built-in data interpretation tools, designed to assist practitioners who may not have an extensive background in data analysis.

Logistical issues with device usage are another barrier, which may include device durability, athlete comfort, or even data transfer efficiency. Technology manufacturers must continue to innovate and improve upon these factors to increase their products’ usability and effectiveness. Feedback from athletes and practitioners should be continually sought and integrated into device design and functionality. Furthermore, the development of wireless technologies, cloud storage, and machine learning algorithms can enhance data transfer efficiency and automate certain aspects of data processing, freeing up more time for coaches and practitioners to focus on data application rather than manipulation. Potential resistance from athletes towards these monitoring devices, whether due to perceived invasion of privacy, discomfort, or scepticism towards the technology’s efficacy, is another challenge that needs to be addressed. It is important to foster an environment of trust and transparency about the purpose and benefits of these tools. Athletes need to understand that the primary goal of using these devices is to enhance their performance and reduce injury risk. Encouraging athlete involvement in the process and providing them with feedback on their progress can enhance buy-in and acceptance of these monitoring tools. Lastly, one cannot overlook the economic barrier that could prevent smaller sports organizations or individual athletes from accessing these technologies. In this regard, the development of cost-effective monitoring devices or alternative affordable solutions will be crucial.

In light of these challenges, it’s clear that the pathway to widespread adoption of these monitoring technologies involves not just technological advancement, but also education, communication, and collaborative efforts. This underlines the importance of continued research and development to enhance the usability, acceptance, and accessibility of these devices in various sports settings. This review provided insights into the current understanding of injury prevention in athletes and identified key themes and gaps in the literature, informing future research in this field. It also emphasized the value of individualized injury prevention strategies tailored to each sport’s specific demands and risk factors. These findings serve as a roadmap for practitioners, coaches, and policymakers in the field of sports science and medicine, outlining the importance of continued research to enhance the safety, performance, and career longevity of athletes globally.

In summarizing the practical utility of these findings, this review underscores the significant role of technologies in sports for both training adaptation and injury prevention. It was highlighted the application of devices such as GPS, accelerometers, and force plates in real-time performance monitoring and risk assessment. These technologies offer valuable insights for coaches and practitioners to tailor training regimens, optimize athlete recovery, and minimize injury risks. This review also points towards an evolving landscape in sports science, where data-driven decisions are becoming increasingly pivotal in enhancing athlete safety and performance.

### Limitations

This scoping review, while thorough, does have inherent limitations that should be considered when interpreting the findings.

First, a formal risk of bias assessment for the included studies was not conducted, which is consistent with the methodology of a scoping review, which primarily seeks to offer a broad overview of existing literature regardless of individual studies’ quality [13]. This means that the methodological quality of the studies included has not been critically appraised, and therefore the findings of this review should be interpreted with caution.

Second, while the aim was to encompass all load measurement devices used in sports, it’s possible that newer technologies or those less frequently used in research may have been underrepresented in the literature reviewed. Consequently, while every effort was made to include all relevant technologies, some may have been inadvertently overlooked.

Finally, the nature of scoping reviews involves drawing broad conclusions from a diverse set of sources. Therefore, the specific contexts, methodologies, and populations of the individual studies were not deeply evaluated, which may limit the applicability of the findings to specific situations or individuals. While the use of load measurement devices has brought valuable insights into athlete performance and injury prevention, caution is warranted in their application due to the heterogeneity in the devices utilized in these studies. Given the varied functionalities and capacities of these devices, their output can differ significantly, potentially influencing the interpretation and application of the data. Therefore, further research is necessary to clarify these results and standardize device usage, ensuring more accurate and reliable data to inform injury prevention strategies in sport. A recommendation for subsequent research would be a more targeted systematic review, focusing on specific devices or sports, to provide more precise insights into the effectiveness of different load measurement strategies.

Despite these limitations, it is believed that this scoping review provides valuable insights into the current understanding of injury prevention in athletes, identifying key themes and gaps in the literature that can guide future research in this field.

## Conclusions

The results of this scoping review reveal a burgeoning area of research that is crucial to injury prevention in athletes across various sports. The evidence demonstrates the prevalent use of diverse devices, including GPS, triaxial accelerometers, gyroscopes, digital compasses, and force plates, to monitor, assess, and consequently mitigate the risk of injuries.

The review has identified a predominant focus on Player Load as the variable of interest in many of the included studies. The different studies have underlined the value of Player Load data in offering specific insights that could guide practitioners in prescribing individualized training loads based on factors such as the nature of the sport, the position of the player, and the physical demands of different drills or competitive situations.

Furthermore, this review also highlighted how other devices, such as force plates, provide critical information, especially in assessing fatigue, facilitating recovery, and guiding injury rehabilitation strategies. The contribution of these tools in evaluating kinetic variables that can influence injury risk is evident, particularly in sports with a high risk of knee and ankle injuries.

Ultimately, this review contributes to a comprehensive understanding of the existing literature on injury prevention in athletes, providing a roadmap for researchers, practitioners, and stakeholders in the sporting realm. It underlines the importance of continued research in this area to enhance the safety, performance, and career longevity of athletes globally.

### Electronic supplementary material

Below is the link to the electronic supplementary material.

Supplementary Material 1

## Data Availability

All data are available upon request to the corresponding author.
